# Erratum to “Validation of a novel claims-based stroke severity index in patients with intracerebral hemorrhage” [J Epidemiol 27 (1) (2017) 24–29]

**DOI:** 10.1016/j.je.2017.04.002

**Published:** 2017-04-06

**Authors:** Ling-Chien Hung, Sheng-Feng Sung, Cheng-Yang Hsieh, Ya-Han Hu, Huey-Juan Lin, Yu-Wei Chen, Yea-Huei Kao Yang, Sue-Jane Lin

**Affiliations:** a Division of Neurology, Department of Internal Medicine, Ditmanson Medical Foundation Chiayi Christian Hospital, Chiayi City, Taiwan; b Department of Neurology, Tainan Sin Lau Hospital, Tainan, Taiwan; c Department of Information Management and Institute of Healthcare Information Management, National Chung Cheng University, Chiayi County, Taiwan; d Department of Neurology, Chi Mei Medical Center, Tainan, Taiwan; e Department of Neurology, Landseed Hospital, Tao-Yuan County, Taiwan; f Department of Neurology, National Taiwan University Hospital, Taipei, Taiwan; g Institute of Clinical Pharmacy and Pharmaceutical Sciences, College of Medicine, National Cheng Kung University, Tainan, Taiwan; h Department of Pharmacy Systems, Outcomes & Policy, College of Pharmacy, University of Illinois at Chicago, Chicago, IL, USA

In our article recently published in the *Journal of Epidemiology*,^1^ the legends of Fig. 1 (p. 26) and Fig. 2 (p. 27) happened to be reversed. They should be corrected as the following:

**Fig. 1.**
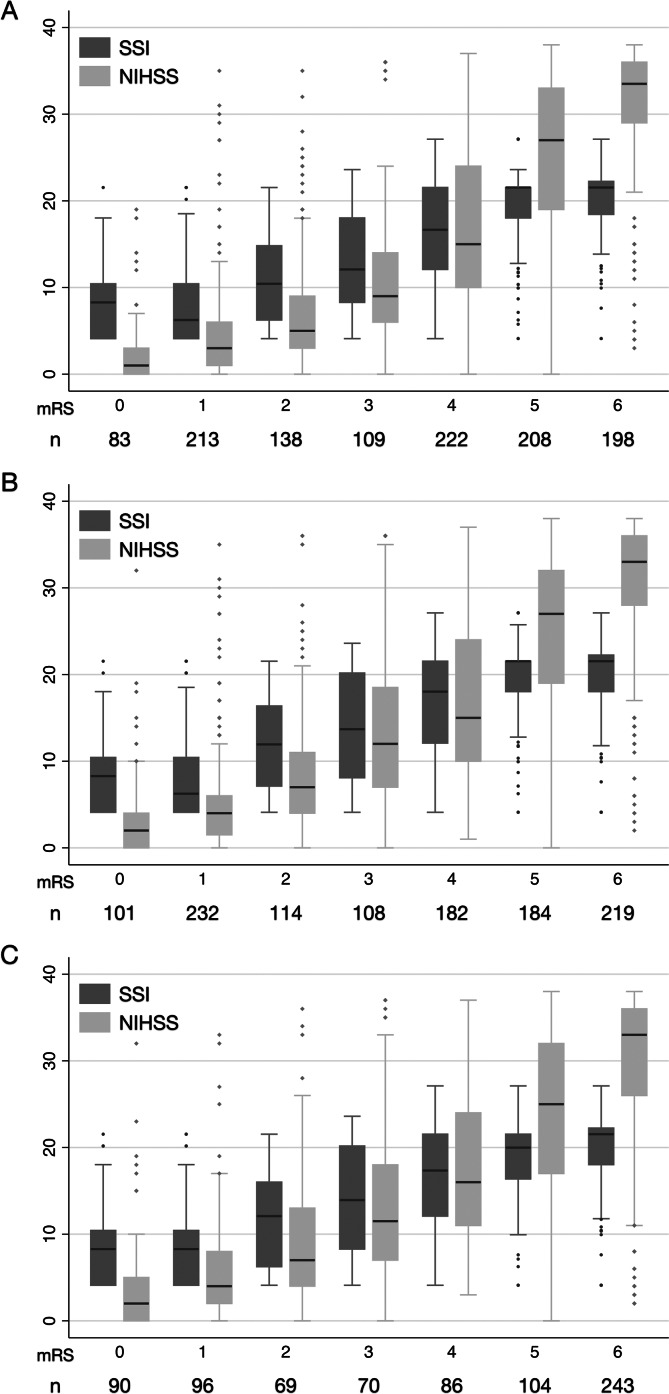
Box-plots showing the distribution of the SSI and the NIHSS across all mRS grades at 3 months (A), 6 months (B), and 1 year (C) after intracerebral hemorrhage. mRS, modified Rankin Scale; NIHSS, National Institutes of Health Stroke Scale; SSI, stroke severity index.

**Fig. 2.**
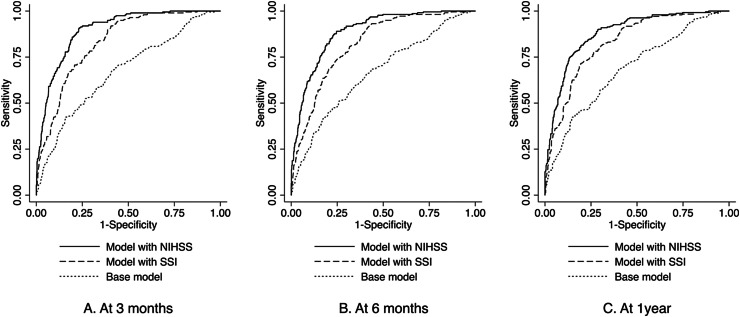
Performance of mortality models for intracerebral hemorrhage. NIHSS, National Institutes of Health Stroke Scale; SSI, stroke severity index.

We sincerely apologize for not being able to identify those errors during the proof reading process.
